# Structure and Bioactivity of a Modified Peptide Derived from the LPS-Binding Domain of an Anti-Lipopolysaccharide Factor (ALF) of Shrimp

**DOI:** 10.3390/md14050096

**Published:** 2016-05-19

**Authors:** Hui Yang, Shihao Li, Fuhua Li, Jianhai Xiang

**Affiliations:** 1 Key Laboratory of Experimental Marine Biology, Institute of Oceanology, Chinese Academy of Sciences, Qingdao 266071, China; victor1900@163.com (H.Y.); lishihao@qdio.ac.cn (S.L.); jhxiang@qdio.ac.cn (J.X.); 2 University of Chinese Academy of Sciences, Beijing 100049, China; 3 Laboratory for Marine Biology and Biotechnology, Qingdao National Laboratory for Marine Science and Technology, Qingdao 266071, China

**Keywords:** LPS binding domain (LBD), antimicrobial, heat stability, structure, cytotoxicity

## Abstract

The lipopolysaccharide binding domain (LBD) in anti-lipopolysaccharide factor (ALF) is the main functional element of ALF, which exhibits antimicrobial activities. Our previous studies show that the peptide LBDv, synthesized based on the modified sequence of LBD (named LBD2) from FcALF2, exhibited an apparently enhanced antimicrobial activity. To learn the prospect of LBDv application, the characteristics of LBDv were analyzed in the present study. The LBDv peptide showed higher antimicrobial and bactericidal activities compared with LBD2. These activities of the LBDv peptide were stable after heat treatment. LBDv could also exhibit *in vivo* antimicrobial activity to *Vibrio harveyi*. The LBDv peptide was found to bind bacteria, quickly cause bacterial agglutination, and kill bacteria by damaging their membrane integrity. Structure analysis showed that both LBDv and LBD2 held the β-sheet structure, and the positive net charge and amphipathicity characteristic were speculated as two important components for their antimicrobial activity. The cytotoxicity of LBDv was evaluated in cultured *Spodoptera frugiperda* (Sf9) cells and *Cherax quadricarinatus* hemocytes. More than 80% cells could survive with the LBDv concentration up to 16 μM. Collectively, these findings highlighted the potential antimicrobial mechanism of LBD peptides, and provided important information for the commercial use of LBDv in the future.

## 1. Introduction

Antimicrobial peptides (AMPs) are evolutionarily conserved small molecules consisting of less than 50 amino acid residues with positive charges and strong amphipathy [1]. They play a major role in the innate immune system as the first line of defense against invading microbes among most multi-cellular organisms [2,3]. These peptides possess a broad spectrum of antimicrobial activity against both Gram-positive and Gram-negative bacteria, fungi, protozoan parasites and some enveloped virus [4,5,6]. AMPs could modulate the innate immune response through a range of activities [7]. The action mechanism of cationic AMPs has been proposed [1]. Usually, the cationic AMPs bind to the negatively charged lipopolysaccharide (LPS) or lipoteichoic acid (LA) of bacteria and then lead to the perforation of their cell walls. Therefore, the cell walls of the bacteria are regarded as the main targets of AMPs [8,9]. Different from traditional antibiotics, AMPs could hardly lead to the bacterial resistance [10]. Therefore, AMPs are regarded as potential candidates of novel antibiotics.

Till now, more and more diverse sequences and structures of AMPs (*i.e.*, α-helical, β-sheet, extended) have been reported in different species. Although diverse sequential and structural variances of AMPs have been documented, most AMPs share some universal physicochemical features [11]. Net cationic charge, hydrophobicity and amphipathicity are the basic characters of active AMPs [12,13]. Based on clarifying the characters of AMPs, more attentions are focusing on the rational design of novel AMPs with the enhanced antimicrobial activity and decreased toxicity against eukaryotic cells by modifying the chain length [14], net charge [15], hydrophobicity [16] and structure [17]. Several novel designed AMPs have been reported through substitution of some amino acids [18,19,20]. The sequence-based approach that correlates antimicrobial activity to the presence of specific amino acid/fragment at specific position is a direct and efficient method among various strategies in designing novel analogues of AMPs [21].

Anti-lipopolysaccharide factors (ALFs) have been regarded as important effectors of the innate immune system in crustaceans [22]. The LPS binding domain (LBD) is considered as the functional domain for antimicrobial and antiviral activities [23,24]. Multiple isoforms of LBDs exhibit different antimicrobial activities against either Gram-positive or Gram-negative bacteria [25,26]. The LBD motif of rALF-*Pm3* shows a β-hairpin structure with two anti-parallel β-strands linking by a disulfide bond [27]. Some synthetic cationic LBDs peptides exhibit antimicrobial activity with high-efficiency by disrupting the bacterial cell wall [28].

Our previous study shows that the synthetic LBD peptide of FcALF2 (named LBD2) from *Fenneropenaeus chinensis* exhibits high inhibition activity to bacteria and white spot syndrome virus (WSSV) [25]. The modified synthetic LBD peptide (designated LBDv) of FcALF2 through substitution of some amino acids shows an apparently enhanced antibacterial activity [29]. In order to learn the prospect of LBDv application in the future, the characters of LBDv were evaluated, and its structure was analyzed to learn the mechanism of increased antimicrobial activity. These data will provide useful information for the development of antimicrobial drugs with high activity.

## 2. Results

### 2.1. Antibacterial and Bactericidal Activity of LBDv Peptides

The minimal growth inhibition concentration (MIC) and the minimal bactericidal concentration (MBC) of LBDv are shown in Table 1. The MIC and MBC of LBDv peptides against both G+ and G− bacteria are much lower than those of LBD2, which indicates that the antibacterial activity of the modified peptide LBDv is enhanced and the antibacterial spectrum is broadened compared with LBD2 peptide without modification.

The MIC and MBC of heat treated LBDv against different bacteria are also shown in Table 1. The MIC and MBC of treated LBDv show no significant difference compared with LBDv without any treatment, suggesting that LBDv shows strong heat stability.

**Figure 1:**
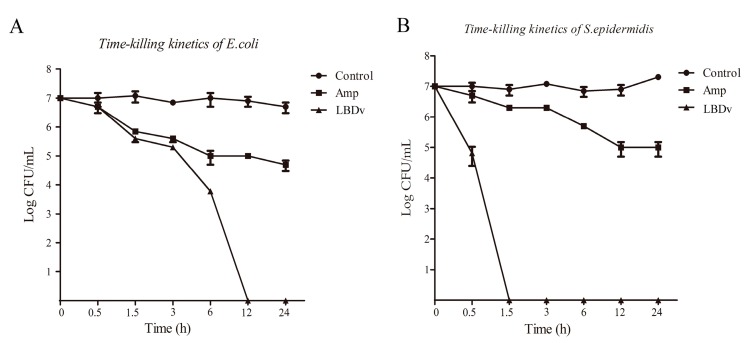
Time-kill experiment of LBDv for *Escherichia coli* and *Staphylococcus epidermidis*. *Y*-axis represents the logarithm of colony forming units (CFU) determined by serial dilution on LB agar. *X*-axis represents the time point after incubation with 64 μM LBDv peptides. The 64 μM ampicillin and pGFP peptides are used as positive and negative control. Data are shown by the means ± S.E. Three replicate experiments are performed.

The time-killing curves of LBDv against *Escherichia coli* and *Staphylococcus epidermidis* are shown in Figure 1. After incubation with 64 μM of LBDv, *E. coli* is completely killed within 12 h, and *S. epidermidis* is killed within 1.5 h. Compared to ampicillin, 64 μM LBDv could kill the target bacteria in much shorter time than ampicillin with same concentration.

### 2.2. In Vivo Detection of the Antimicrobial Activity of LBDv Peptide

**Figure 2:**
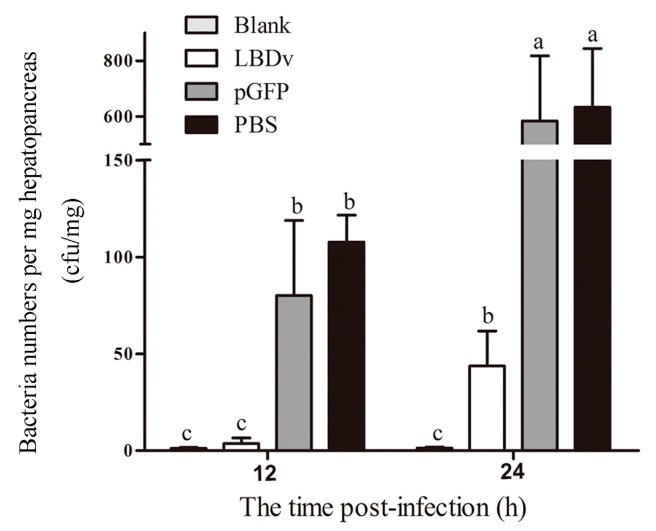
*In vivo* effect of LBDv on bacterial infections. Data are shown as the means ± S.E. Colony forming units of *Vibrio harveyi* are assessed after plating samples of the hepatopancreas onto TCBS agar plates. Lowercase letters (a, b, and c) represent significant difference among treatments at *p* < 0.05. The data are analyzed based on ANOVA with *post hoc*.

The colony numbers of *Vibrio harveyi* in the hepatopancreas of shrimp at 12 h and 24 h after injection are shown in Figure 2. The colony numbers of *V. harveyi* in the hepatopancreas of shrimp from LBDv peptide group are significantly lower (*p* < 0.05) than those in pGFP and PBS group at both 12 h and 24 h after injection.

### 2.3. The Binding and Agglutination Activity of LBDv

**Figure 3:**
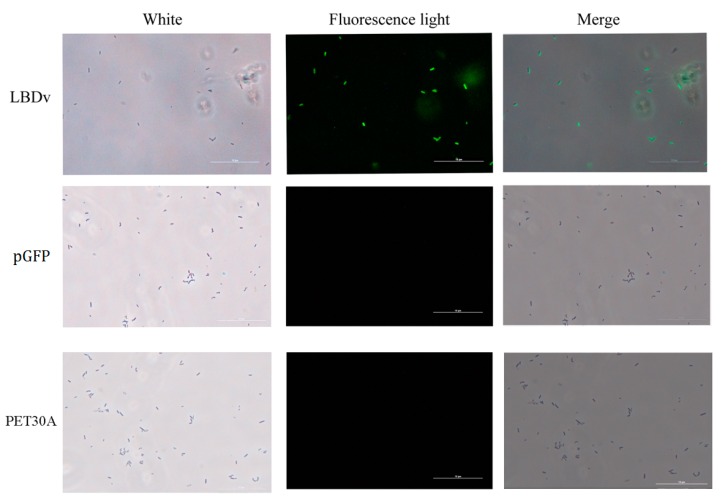
Interaction of LBDv-His with *E. coli* bacterial cells. The signal is detected by immunocytochemistry with anti-His polyclonal antibody as the primary antibody. The same concentration of pGFP peptide and PET30A are used as negative controls. Scale bar is 10 μm.

The binding detection of LBDv-His peptide to *E. coli* is shown in Figure 3. Compared with the control, apparent fluorescence is observed for *E. coli* after incubation with the LBDv-His peptide, whereas no fluorescence is detected in the controls (pGFP and PET30A treatments).

**Figure 4:**
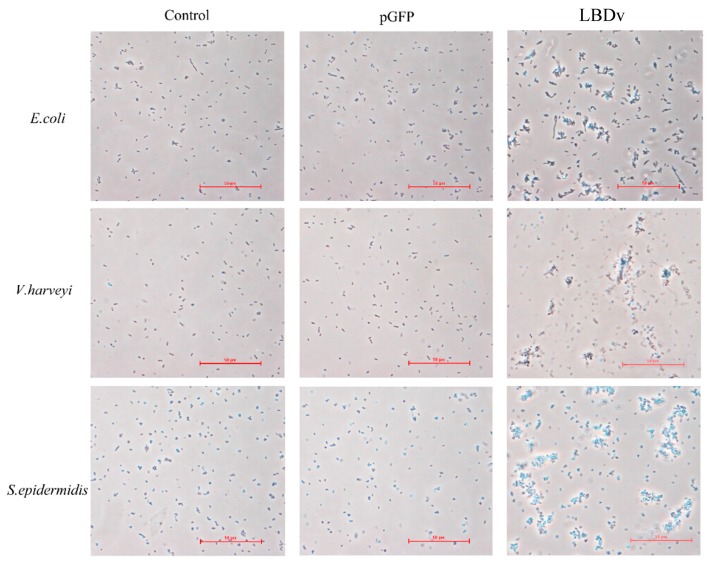
Detection of bacterial agglutination experiment. Three kinds of bacteria are selected, including *E. coli*, *V. harveyi* and *S. epidermidis*. The 10^5^ cfu/mL different bacteria are incubated with 64 μM LBDv and pGFP peptides within 30 min, respectively, and observed under optical microscope. Scale bar is 50 μm.

Agglutination detection shows that LBDv peptide could lead to apparent agglutination of the bacteria *E. coli*, *V. harveyi*, and *S. epidermidis* (Figure 4).

**Figure 5:**
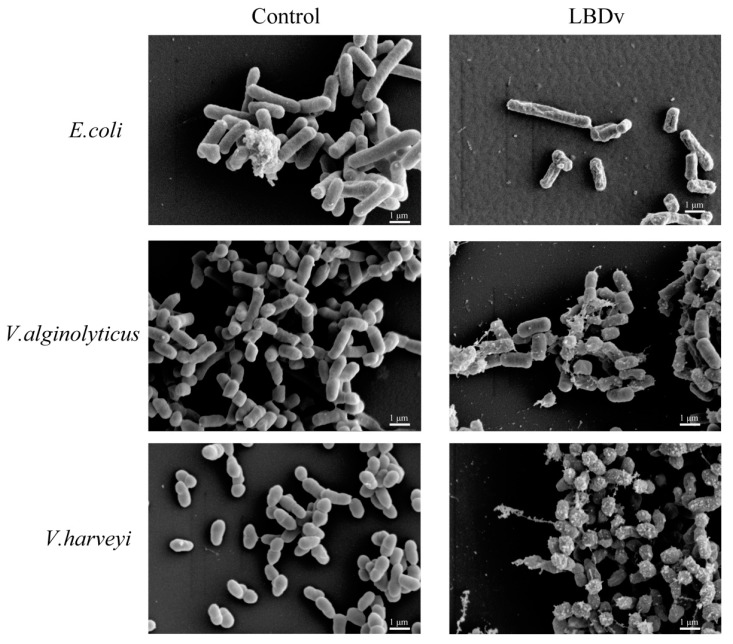
Morphology of the bacteria after treatments by LBDv peptide. The 10^8^ cfu/mL different bacteria are incubated with 64 μM LBDv peptide for 2 h. The bacteria treated with same concentration pGFP peptide are used as control. Scale bar is 1 μm.

When we observe the bacteria after incubation with LBDv peptide under scanning electronic microscope, we find that the surface of bacteria treated with LBDv peptide is severely damaged with leakage of some cytoplasm, while the bacteria in control group show a relatively smooth surface (Figure 5).

### 2.4. Structure Analysis of LBDv Peptide

**Figure 6:**
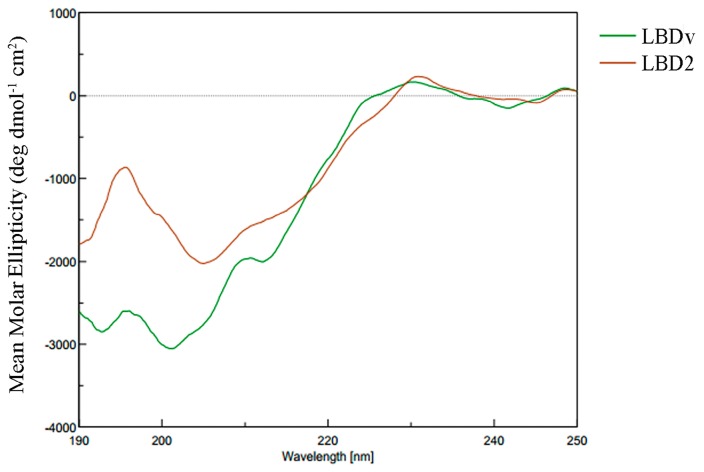
The CD spectra of LBD2 and LBDv peptide. The peptide concentration is 0.2 mg/mL.

CD spectroscopy is used to measure the secondary structure of LBD2 and LBDv peptides in 20 mM phosphate buffer (pH 7.0). The CD spectra of the peptides are shown in Figure 6, which indicates that LBDv and LBD2 show a very similar secondary structure. Further analysis by Protein SSE software shows that the spectra of LBD2 and LBDv are characteristic of β-sheet conformations. The structure components of LBDv and LBD2 are shown in Table 2. LBD2 consists of 79.7% β-sheet and 20.3% random coil, while LBDv consists of 71.5% β-sheet, 4.1% β-turn and 24.4% random coil.

**Figure 7:**
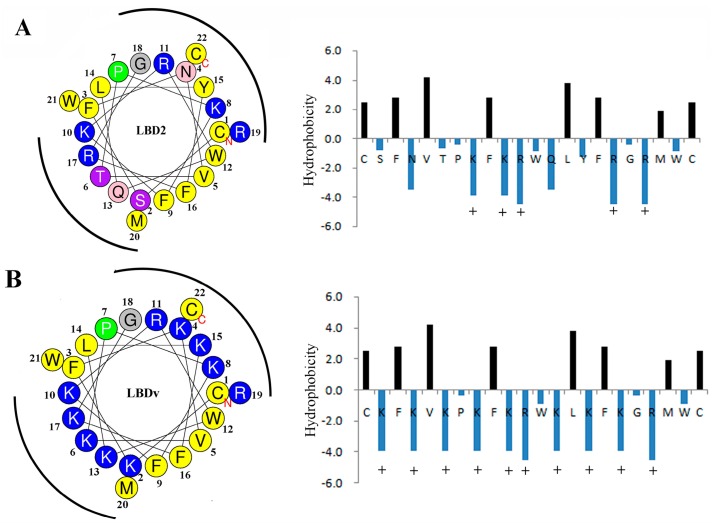
Helical wheel prediction and β-strand diagrams showing the distribution of amino acid side chains: (**A**) LBD2; and (**B**) LBDv. + means Lysine or Arginine. The blue color represents basic amino acids, yellow color represents the hydrophobic residues, and the other colors present the hydrophilic residues with different hydrophilic abilities.

The physicochemical properties and amphipathic characters of LBDv and LBD2 are further analyzed by ProtParam tool and HeliQuest analysis. The wheel diagram and β-strand diagrams (Figure 7) show that the positively charged hydrophilic amino acid residues of LBDv are located on two sides, whereas the hydrophobic residues are on the other two sides, presenting a perfect amphipathic structure for both LBD2 peptide and LBDv. The physiochemical parameters of LBD2 and LBDv are shown in Table 3. The positive net charge and hydrophilicity of LBDv are significantly higher than those of LBD2 peptide.

### 2.5. Evaluation on the Cytotoxicity of LBDv Peptide

**Figure 8:**
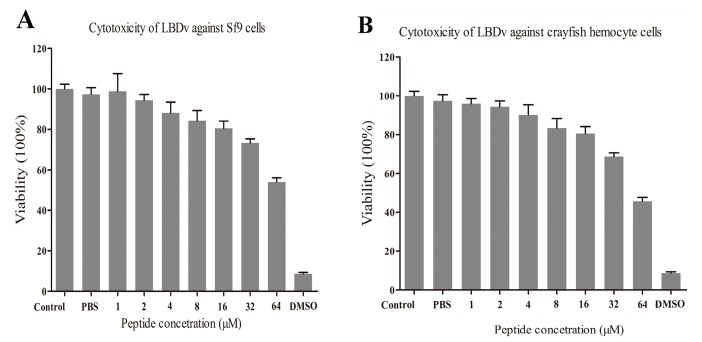
MTT assay of Sf9 cells and crayfish hemocytes at different LBDv concentrations: (**A**) cytotoxicity of LBDv against Sf9 cells; and (**B**) cytotoxicity of LBDv against crayfish hemocytes.

Sf9 cells and primary cultured cells of *Cherax quadricarinatus* hemocytesare used for the cytotoxicity detection and the cell viability is detected after addition of different concentration of peptides. From the results (shown in Figure 8), more than 80% of Sf9 cells and *C. quadricarinatus* hemocytes could survive in the LBDv solution with a concentration up to 16 μM. High concentration LBDv peptides solution has toxic effects on both Sf9 cells and hemocytes. Above 32 μM, LBDv exhibits significant toxicity on both cells (with mortalities of ~30% at 32 μM and ~50% at 64 μM).

## 3. Discussion

Antibiotic resistance is one of the main problems concerning public health or clinical practice. AMPs have attracted a great deal of attention as the alternative antibiotic candidates [30]. The LBD peptides of ALF with 22 amino acid residues and a disulfide bond share some common traits with other AMPs [25,31]. In our previous study, a modified peptide (LBDv) derived from the LBD domain of ALF shows an enhanced antimicrobial activity, but its biological characteristics are not clear. In the present study, the biological characteristics of LBDv were analyzed. LBDv peptide could kill both Gram-negative bacteria *E. coli* and Gram-positive bacteria *S. epidermidis* at short time as other AMPs like defensin [32]. The LBDv peptide could bind to bacteria directly, which is in accordance with previous reports that LBD peptide of ALF shows a high affinity to lipopolysaccharide (LPS) and lipoteichoic acid (LA) [33]. It is interesting to note that both Gram-negative and Gram-positive bacteria could be agglutinated quickly by LBDv peptide. This phenomenon is rarely reported for AMPs, except for thanatin from insects [34]. In general, the binding of cationic peptide could reduce the surface charge density and thus reduce the electrostatic repulsion between bacteria, allowing their aggregation. Through observation on the surface of the bacteria, we found that LBDv peptide could disrupt the bacteria membrane and then cause the leakage of cytoplasm from the SEM detection. This might be the reason that LBDv peptide functions to kill bacteria. Till present, a variety of models for antimicrobial mechanism have been proposed, including the carpet, barrel-stave pore, toroidal pore, and aggregate models [35,36,37]. The proper model suitable for LBDv still needs further investigation.

Even though many naturaland modified AMPs have been studied with considerable advantages for therapeutic applications, there are still some limitations for drug development. Many AMPs generally have low stability *in vivo* due to the complex surrounding environments, such as the presence of protease, pH change, and physiological salt and serum conditions [38,39,40]. In the present study, the engineered LBDv peptide exhibits apparent *in vivo* antimicrobial activity to *V. harveyi*. *V. harveyi* has been considered as a pathogenic agent causing massive mortality in shrimp industry [41]. The effect of inhibition to this pathogenic microbe *in vivo* by LBDv peptide could provide some new strategies for shrimp disease control.

The secondary structure of LBDv and LBD2 was compared by CD spectra. LBDv and LBD2 sharevery similar structure such as a β-sheet. Therefore, we propose that the higher antimicrobial activity of LBDv than LBD2 to bacteria might be caused by variation of other elements. Evidence have supported that physicochemical properties of AMPs, rather than any specific amino acid sequence, are responsible for their microbiological activities [42]. Through analyzing the positive net charge and hydrophilicity of LBDv and LBD2, we found that LBDv had higher positive net charge and hydrophilicity than LBD2. The net charge of cationic AMPs can modulate antimicrobial specificity and efficacy of these peptides [43]. The cationicity of AMPs is considered to be beneficial for the initial electrostatic interactions between the AMPs peptides and negatively charged bacterial membrane components [44,45]. Increasing the net charge from +4 to +5 for magainin 2 could increaseits antimicrobial activity [15]. Giangaspero *et al.* shows that decreasing the net charge could reduce the antimicrobial activity of AMPs [46]. Besides the net charge, it is well accepted that the amphipathicity of AMPs is necessary for their mechanism of action [47,48]. Amphipathicity with the positively charged polar face and the non-polar face is required for insertion into the membrane interface, causing increased permeability and loss of barrier function of target cells [49]. The rational distribution of hydrophilic and hydrophobic residues for the LBDv peptides might be the reason for the enhanced antimicrobial activity.

## 4. Materials and Methods

### 4.1. Peptides Synthesis

According to our previous study, we synthesized a modified peptide (LBDv) with sequence of Ac-YCKFKVKPKFKRWKLKFKGRMWCP-NH_2_ based on the LBD of FcALF2 with sequence of Ac-YCSFNVTPKFKRWQLYFRGRMWCP-NH_2_ (accession number: JX853775) [29]. The LBDv peptide was modified with increasing the number of basic amino acids by replacing six residues (S, N, T, Q, Y and R) with lysine (K) residues. A pGFP peptide from Green Fluorescent Protein with sequence of Ac-TTGKLPVPWPTLVTTFSYGVQCFS-NH_2_ was also synthesized as negative control. Different peptides including LBDv, LBD and pGFP were produced by solid-phase synthesis by Ziyu Biotechnology Company (Shanghai, China). Synthetic peptides were characterized and purified by reversed-phase high-pressure liquid chromatography with >95% purity grade.

### 4.2. Antibacterial Activity Test

The antimicrobial activity of peptides was determined using the minimal growth inhibition concentration (MIC) assay and minimal bactericidal concentration (MBC) assay against Gram-positive and Gram-negative bacteria as described previously [25,26]. Bacteria strains including four Gram-negative bacteria, *Escherichia coli*, *Vibrio alginolyticus*, *Vibrio harveyi*, and *Vibrio parahaemolyticus,* and three Gram-positive bacteria, *Bacillus licheniformis, Staphylococcus epidermidis* and *Micrococcus luteus*, were used for detection. Briefly, bacteria cells at the mid-logarithmic-phase were diluted to 1 × 10^5^ cfu/mL in PBS buffer (137 mmol/L NaCl, 2.7 mmol/L KCl, 10 mmol/L Na_2_HPO_4_, 1.8 mmol/L KH_2_PO_4_, pH 7.4). In sterile 96-well plates (Corning Inc., Corning, NY, USA), 15 μL peptide solution in 1/2-fold serial dilutions with PBS (pH 7.4) were added into each well. The final concentration of peptide in the medium was 64 μM, 32 μM, 16 μM, 8 μM, 4 μM, 2 μM, 1 μM and 0.5 μM. Fifteen microliters PBS and 15 μL pGFP solution were used as blank group and negative control. Then, 133 μL growth medium were added into the 96-well plates, and the mixtures were allowed to culture for 6 to 8 h depending on different bacterial strains. Absorbance at 600 nm for Gram-positive bacteria or 560 nm for Gram-negative bacteria was determined using a precision micro-plate reader (TECAN infinite M200 PRO, Salzburg, Austria). The assay was performed in triplicates. The MICs were defined as the lowest concentration of the compounds to inhibit the growth of microorganisms based on the spectroscopic absorbance readings. MBCs were determined by plotting 5 μL samples from the wells onto nutrient agar plate. MBC was the concentration at which there was no microbial growth [50]. Because only 8 peptides concentration gradients were set, the MICs and MBCs value observed at the concentration point were higher than the actual values. Thus, the actual and accurate MICs and MBCs value should between no inhibition (or bactericidal) concentrations and the observed inhibition (bactericidal) concentrations.

In order to know the heat tolerance of the peptide, the peptide LBDv was treated at 100 °C for 10 min, and its MIC and MBC value was measured as described above. The assay was performed in triplicates.

### 4.3. The Time-Killing Curve against Bacteria

The procedure used for the kinetics of bacterial killing assays was modified as previously described [51] to determine the rate of bacterial killing by LBDv peptide. Bacteria *E. coli* and *S. epidermidis* were chosen for the experiment. *E. coli* and *S. epidermidis* cells were grown in LB medium to exponential phase, harvested by centrifugation and washed with PBS, then bacteria was resuspended in PBS buffer. Approximately 1 × 10^8^ cells were incubated at 37 °C with 64 μM peptide. Aliquots of 20 μL suspensions were withdrawn, serially diluted and plated on LB agar to determine the bacterial counts at 0, 0.5, 1.5, 3, 6, 12 and 24 h. After culturing at 37 °C, the number of bacteria was recorded. The time-killing curves were plotted with the log cfu/mL against time. The 64 μM ampicillin and pGFP peptides were used as positive and negative control. Triple independent experiments were performed for time-killing kinetics.

### 4.4. Effects of the Peptides on Bacterial Infections

For bacterial infection, *V. harveyi* was cultured in TSB medium at 28 °C overnight. Then bacteria cells were resuspended in sterile PBS to 5 × 10^7^ cfu/mL. pGFP peptide and LBDv peptide were added to the cells to the final concentration of 32 μM. The bacteria diluted by PBS were used as the negative control. *Exopalaemon carinicauda* with body weight of 0.68 ± 0.17 g were used as the experimental animals and divided randomly into four groups: Blank, LBDv, pGFP and PBS group. The three experiment groups were injected intramuscularly with 10 μL *V. harveyi, V. harveyi* plus LBDv, or *V. harveyi* plus pGFP, respectively. Hepatopancreas was taken from shrimp at 12 h and 24 h post-infection. Three samples were collected from each group at each time point, and three individuals were put togetheras one sample. The tissues were homogenized in sterile PBS, serial diluted and plated in triplicate on TCBS agar plates. After incubation at 28 °C for 24 h, the number of colonies on the plates was recorded.

### 4.5. Bacteria Binding Assay

To analyze the binding activity of LBDv peptide to bacteria, the peptides with a 6× His tag have been synthesized as described previously. The *E. coli* cells were cultured in LB medium overnight at 37 °C. The bacteria were then collected by centrifugation, washed in PBS buffer (pH 7.4), and resuspended in PBS buffer to 10^8^ cfu/mL. Then, 200 μL bacteria cells were incubated with 64 μM LBDv-His protein for 2 h at room temperature, then bacteria suspension was dropped onto a glass slide and fixed with 4% paraformaldehyde for 15 min, followed by washing with PBS. After treatment in blocking solution (0.5% BSA in PBS), mouse anti-His antibody (Tiangen, Beijing, China) (1/1000 dilution) was added to the slide.The slide was incubated for 1 h at room temperature and washed with PBS. Fluorescein isothiocyanate (FITC)-labeled goat anti-mouse IgG (1/1000 dilution) was added to the slide. The slide was incubated and washed as above, and observed under a fluorescence microscope (Nikon E800, Tokyo, Japan). pGFP peptide was used as control. At the same time, to exclude His tag’s influence on the bacteria binding, we also add the protein with 6×His tag produced by empty vector pET30a in *E. coli* (named PET30A protein) as negative control.

### 4.6. Bacterial Agglutination Experiment

The Gram-negative bacteria (*E. coli* and *V. harveyi*), and Gram-positive bacteria (*S. epidermidis*) were cultured overnight. Then, bacteria cells were diluted to 10^5^ cfu/mL. After that, 64 μM LBDv peptide solution was incubated with the bacteria at room temperature within 30 min. The PBS and 64 μM pGFP peptide solution were used as negative control. The cells were then added to the glass slide and observed under optical Nikon TS100 microscope (Nikon Corporation, Tokyo, Japan).

### 4.7. Morphology of the Bacteria Treated with LBDv

The bacteria *E. coli*, *V. alginolyticus* and *V. harveyi* were grown to an exponential phase with shaking at 220 rpm. The cell pellets were harvested by centrifugation at 1000× *g* for 10 min and resuspended in PBS at 10^8^ cfu/mL. Bacteria were incubated with 64 μM different peptides at 37 °C for 2 h. The collected bacteria were subjected to fixation with 2.5% (*v*/*v*) glutaraldehyde in 0.1 M phosphate buffer (pH 7.4) for 1 h and dehydrated for 15 min in a graded ethanol series (30%, 50%, 70%, 80%, 95% and 100%), and a mixture (1:1) of 100% ethanol and isopentyl acetate for 20 min. After critical-point drying and gold coating, the samples were visualized by Hitachi S-3400N Scanning Electron Microscope (Hitachi, Tokyo, Japan).

### 4.8. Structure Analysis on the Peptide LBDv and LBD2

The secondary structure content of LBD2 and LBDv were measured by circular dichroism spectroscopy (JASCO 715, Tokyo, Japan) in far UV. Spectra were recorded at room temperature, over the range of 190 to 250 nm. The peptide concentration was 0.2 mg/mL in 20 mM phosphate buffer pH 7.0. We used a scan speed of 50 nm/min, a 0.1 cm cell path length, a response time of 4 s and a band width of 1 nm. A blank spectrum containing all components except the peptide was subtracted from individual spectra to account for the baseline. Finally, the data were smoothed using Jwstda32 software. The acquired CD spectra were then converted to the mean molar ellipticity (deg·dmol^−1^·cm^2^). The secondary structural elements of the peptides, including α helix, β-sheet, β-turn and random coil, were calculated based on the CD spectra using Protein SSE software according to a standard reference spectrum.

Different physicochemical properties of peptides (*i.e.*, net charge and hydrophobic percentage) were calculated using the online program Antimicrobial Peptide Database (http://aps.unmc.edu/AP/main.php) and ProtParam tool (http://web.expasy.org/protparam/). The amphipathic characters of these peptides were predicted using the online program HeliQuest analysis (http://heliquest.ipmc.cnrs.fr/cgi-bin/ComputParams.py).

### 4.9. Cytotoxicity Assays

Cytotoxicity against *Spodoptera frugiperda* (Sf9) cell lines and *Cherax quadricarinatus* hemocytes were examined by a tetrazolium-based (MTT) assay. The Sf9 cell lines were routinely maintained at 27 °C in sterile 48-well plates (Corning Inc., Corning, NY, USA) containing 200 μL Grace’s insect cell culture medium (Gibco Inc., Grand Island, NY, USA). Hemolymph was taken from *C. quadricarinatus* with equal volume of anticoagulant solution (26 mM sodium citrate, 30 mM citric acid, 100 mM glucose, 140 mM NaCl, pH 5.8). Then hemocytes were collected by centrifugation at 800 g for 5 min and resuspended in L15 medium (Gibco Inc., Grand Island, NY, USA) supplemented with 15% fetal bovine serum. The hemocytes were seeded in sterile 48-well plates (Corning Inc., Corning, NY, USA) with a density of 1 × 10^5^ cells/well and maintained at 27 °C. For the MTT assay, sterile peptide solution with PBS (pH 7.4) were added into each well to the final concentration of 1, 2, 4, 8, 16, 32, 64 μM. DMSO (0.5%) was added into the medium as the positive control. Triple independent experiments were performed. After 24 h, the cells were washed with PBS and treated with MTT formazan at a concentration of 0.5 mg/mL. After 6 h, crystal was centrifuged and dissolved in dimethyl sulfoxide, and the absorbance was determined at 570 nm using a precision micro-plate reader (TECAN infinite M200 PRO, Salzburg, Austria) to determine the percent cytotoxicity.

### 4.10. Statistical Analysis

All the data were given in the form of mean ± S.E. and analyzed with one-way analysis of variance (ANOVA) and Duncan’s Multiple Comparisons. Differences between treatments and controls were considered significant at *p* < 0.05.

## 5. Conclusions

In conclusion, the engineered peptide LBDv exhibits broad-spectrum antimicrobial and antiviral activities *in vitro* and *in vivo*. We propose that net positive charge and amphipathicity characteristic are the important components for antimicrobial activity of different LBDs. Further cytotoxicity analysis of LBDv peptide suggests that the peptide has application prospect. These data provide useful information to develop new AMPs drugs for potential therapeutic application in medical areasas well as in control of disease in aquaculture.

## Figures and Tables

**Table 1:** Antimicrobial activities of the engineered LPS-binding domain (LBD) peptides after incubation with different kinds of bacteria

Microorganisms	LBD2 (μM)		LBDv (μM)		Heated LBDv (μM)	
	MIC ^a^	MBC ^b^	MIC	MBC	MIC	MBC
Gram negative bacteria (G−):						
*Escherichia coli*	>64	>64	0.5–1	4–8	1–2	4–8
*Vibrio alginolyticus*	2–4	16–32	1–2	8–16	2–4	8–16
*Vibrio harveyi*	2–4	16–32	1–2	2–4	2–4	2–4
*Vibrio Parahaemolyticus*	32–64	32–64	1–2	4–8	2–4	4–8
Gram positive bacteria (G+):						
*Bacillus licheniformis*	16–32	>64	8–16	32–64	16–32	32–64
*Staphylococcus epidermidis*	32–64	>64	4–8	16–32	4–8	16–32
*Micrococcus luteus*	2–4	>64	4–8	16–32	4–8	16–32

^a^ MIC, minimal inhibitory concentration; ^b^ MBC, minimal bactericidal concentration.

**Table 2:** Secondary content structure of LBD2 and LBDv peptides calculated using Protein SSE software based on the CD spectra.

Peptide	Structure Content (%)			
	α-helical	β-sheet	β-turn	Random coil
LBD2	0	79.7	0	20.3
LBDv	0	71.5	4.1	24.4

**Table 3:** Key physicochemical parameters of LBD2, LBDv and pGFP peptides.

Peptide	Sequence	NetC ^a^	H ^b^	HR (%) ^c^	GRAVY ^d^	μH ^e^
LBD2	Ac-Y(CSFNVTPKFKRWQLYFRGRMWC)P-NH_2_	5	0.619	41	−0.6042	0.103
LBDv	Ac-Y(CKFKVKPKFKRWKLKFKGRMWC)P-NH_2_	10	0.398	41	−0.9409	0.072
pGFP	Ac-TTGKLPVPWPTLVTTFSYGVQCFS-NH_2_	1	0.732	37	0.3333	0.314

^a^ NetC means net charge. Lys (K), Arg (R), His (H), and C-terminal amidation were assigned with +1 charge. Asp (D) and Glu (E) were assigned with −1 charge; ^b^ H represents mean hydrophobicity that is derived from dividing total hydrophobicity (sum of hydrophobicity indices of all residue) by the number of residues; ^c^ HR means hydrophobic ratio; ^d^ GRAVY means the Grand Average hydropathy value of the peptide; ^e^ μH represents mean hydrophobic moment.
